# Evaluation of autoantibodies as predictors of treatment response and immune‐related adverse events during the treatment with immune checkpoint inhibitors: A prospective longitudinal pan‐cancer study

**DOI:** 10.1002/cam4.4675

**Published:** 2022-03-16

**Authors:** Dominik A. Barth, Stefanie Stanzer, Jasmin Spiegelberg, Thomas Bauernhofer, Gudrun Absenger, Florian Posch, Rainer Lipp, Michael Halm, Joanna Szkandera, Marija Balic, Armin Gerger, Maria A. Smolle, Georg C. Hutterer, Christiane Klec, Philipp J. Jost, Julia Kargl, Martin Stradner, Martin Pichler

**Affiliations:** ^1^ Division of Oncology Department of Internal Medicine, Medical University of Graz Graz Austria; ^2^ Division of Haematology Department of Internal Medicine, Medical University of Graz Graz Austria; ^3^ Department of Orthopaedics and Trauma Medical University of Graz Graz Austria; ^4^ Department of Urology Medical University of Graz Graz Austria; ^5^ Otto Loewi Research Center Division of Pharmacology, Medical University of Graz Graz Austria; ^6^ BioTechMed‐Graz Graz Austria; ^7^ Division of Rheumatology Department of Internal Medicine, Medical University of Graz Graz Austria; ^8^ Department of Experimental Therapeutics The University of Texas MD Anderson Cancer Center Houston Texas USA

**Keywords:** autoimmunity, cancer, immune checkpoint inhibitor therapy, immune‐related adverse events, monoclonal antibodies

## Abstract

**Background:**

The presence of autoantibodies in the serum of cancer patients has been associated with immune‐checkpoint inhibitor (ICI) therapy response and immune‐related adverse events (irAEs). A prospective evaluation of different autoantibodies in different cancer entities is missing.

**Materials and Methods:**

In this prospective cohort study, we included a pan‐cancer cohort of patients undergoing ICI treatment and measured a comprehensive panel of autoantibodies at treatment start and at the time point of first response evaluation. The presence and induction of autoantibodies (ANA, ENA, myositis, hepatopathy, rheumatoid arthritis) in different cancer entities were assessed and the association between autoantibodies and disease control rate (DCR), objective response rate (ORR), and progression‐free survival (PFS), as well as the development of grade 3 or higher irAEs were evaluated by logistic regression models, cox proportional hazard models, and Kaplan–Meier estimators.

**Results:**

Of 44 patients with various cancer entities, neither the presence of any positive autoantibody measurement nor the presence of positive antinuclear antibodies (ANA) [≥1:80] at baseline was associated with the examined clinical endpoints (DCR, ORR, PFS) in univariable and multivariable analyses. After 8–12 weeks of ICI treatment, DCR, ORR, and PFS did not significantly differ between patients with and without any positive autoantibody measurement or positive ANA titers. The frequency of irAEs did not differ depending on autoantibody status of the patients.

**Conclusion:**

Autoantibodies at treatment initiation or induction after 8–12 weeks of ICI treatment are not associated with treatment efficacy as indicated by DCR, ORR, and PFS or higher grade irAEs.

## Graphical abstract

Autoantibodies at treatment initiation or induction after 8‐12 weeks of ICI treatment are not as‐sociated with treatment efficacy as indicated by DCR, ORR and PFS or higher grade irAEs in a prospective longitudinal study.

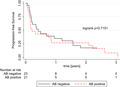

## 1 INTRODUCTION

The development and successful implementation of immune checkpoint inhibitors (ICIs) over the recent years represented a major leap forward for antineoplastic therapy.^1^ By targeting various immune checkpoint molecules, such as programmed cell death protein 1 (PD‐1), programmed cell death 1 ligand 1 (PD‐L1), and cytotoxic T‐lymphocyte‐associated protein 4 (CTLA4) with monoclonal antibodies, T‐cells are enabled to develop specific capabilities to overcome cancer immune evasion and thus eventually target cancer cells.^2^ The introduction of PD‐L1‐ and CTLA4‐inhibitors in the treatment of malignant melanoma a couple of years ago has revolutionized survival outcomes^3^; since then ICIs gained an important role and became a promising treatment option for several cancer entities, including lung,^4^, ^5^ urothelial,^6^ renal,^7^, ^8^ head and neck,^9^ as well as breast cancer.^10^ More recently, ICIs have also been established in neo‐adjuvant/adjuvant treatment settings of various cancer entities.^11^, ^12^

Despite a certain number of patients responding well to ICIs treatment even with a considerable rate of complete‐ (CR) and long‐lasting remissions (depending on cancer type), the majority of patients do not seem to benefit from immunotherapy.^2^ Several biomarkers, such as the tumor mutational burden, PD‐L1 expression of tumor cells, or scores like the combined positive score have been suggested to be able to better select patients and to improve the prediction of individualized ICI treatment efficacy.^13^, ^14^ However, these biomarkers are not able to perfectly predict treatment success in any given case and therefore even patients with high PD‐L1 expressing tumors might experience treatment failures. Besides their financial burden,^15^ ICIs carry a risk of serious, sometimes even life‐threatening immune‐related side effects. Thus, the identification of reliable biomarkers to predict oncological treatment responses as well as immune‐related toxicities is of paramount importance.

Dangerous autoimmune reactions, such as hypophysitis, colitis, or pneumonitis rank among the most significant adverse effects of ICI treatments, which may even lead to the necessity of permanent therapy discontinuation.^16^ Routinely assessable biomarkers to identify patients who carry a highly increased risk for the development of immune‐related adverse events (irAEs), have not entered daily clinical practice yet.^17^ The occurrence of irAEs may be accompanied by the emergence of different autoantibodies in the serum of patients, such as antinuclear (ANA) or extractable nuclear antigen (ENA) antibodies.^18^, ^19^ Thus, autoantibodies were previously discussed as potential biomarkers for the occurrence of irAEs during ICI treatment.^20^ Recently, several retrospective studies indicated that preexisting autoantibodies might be able to predict responses and survival outcomes of ICI‐treated patients, and, moreover may also be associated with an elevated risk for the development of irAEs.^18^, ^21^, ^22^, ^23^ However, other current studies reported controversial results and are mainly focused on non‐small cell lung cancer (NSCLC) patients.^19^, ^23^, ^24^, ^25^ The aim of this study is to characterize the association of autoantibody presence, ICIs‐associated autoantibody induction, treatment response, and irAEs in a prospective pan‐cancer cohort.

## 2 MATERIALS AND METHODS

Forty‐five consecutive cancer patients who were treated at the Division of Oncology, Department of Internal Medicine, Medical University of Graz and received ICI therapy between 2017 and 2020, were included in this prospective longitudinal biomarker study. Patients who were older than 18 years of age, had metastatic or locally advanced solid cancer and received ICI treatment were included in the study. Pretreatment was allowed and patients with preexisting autoimmune diseases of any kind were excluded from the study. One patient was excluded due to loss of follow up, thus 44 consecutive cancer patients entered the final analysis.

Patients had first blood draw at the date before treatment initiation and a second one 8–12 weeks after initiation of ICI treatment. Patients were visited by an experienced oncologist before each treatment administration and each time laboratory assessments for the detection of irAEs included liver parameters (ALT, AST, GGT, Bilirubin), kidney parameters (creatinine, eGFR), muscle enzymes (creatinine kinase), lipase, as well as endocrine parameters (including TSH, fT3, fT4, cortisol, and ACTH) were made. Adverse events were graded according to the Common Terminology Criteria for Adverse Events version 5. Patients were evaluated for treatment response every 8–12 weeks by CT or MRI scans as appropriate, considering RECIST version 1.1 criteria.

Antinuclear antibodies were examined by indirect immunofluorescence on Hep2 cells according to the international consensus and nomenclature.^26^ Antibodies to extractable nuclear antigens (ENA) (anti‐centromere protein B (CENPB), anti‐double strand DNA (dsDNA), anti‐La, anti‐PM100, anti‐PM75, anti‐RNP70, anti‐Ro, anti‐SCL70, anti‐U1RNP, and anti‐cyclic citrullinated peptide antibodies (ACPA)) were analyzed by fluorescence immunoassay (allThermo Fisher) using an automated fluorescence reader (Phadia 250, Thermo Fisher). Rheumatoid factor IgA was measured by ELISA (Orgentec Diagnostika). Anti‐GP210, anti‐LKM1, anti‐M2, anti‐SP100, anti‐SLA‐LP, anti‐LC1, anti‐F‐Actin (LIVER PROFILE 7 Ag DOT, Alphadia) and anti‐EJ, anti‐JO1, anti‐Ku, anti‐MDA5, anti‐MI2a, anti‐MI2b, anti‐NXP2, anti‐Oj, anti‐PL‐12, anti‐PL‐7, anti‐SAE, anti‐SRP, anti‐TIF‐1γ (EUROLINE Autoimmune Inflammatory Myopathies, Euroimmun) were analyzed using immunodot assays. Automated readout according to the manufacturer's protocol yielded semiquantitative results.

### 2.1 Statistical analyses

Disease control rate (DRC), defined as the rate of patients who experienced CR, partial remission (PR), or stable disease (SD), and objective response rate (ORR), defined as the rate of patients who had CR or PR, were considered as co‐primary endpoints of this study. Secondary endpoints were progression‐free survival (PFS), defined as the time from treatment onset to the date of disease progression or death of any cause, and the development of grade 3 or higher irAEs. Autoantibodies were categorized into ANA, ENA, rheumatoid arthritis, hepatopathy, and myositis autoantibodies. Patients were considered antibody positive if at least one autoantibody titer showed results higher than the upper limit of normal (ULN). Patients were considered ANA positive if ANA titers were ≥1:80.

To assess the association of clinicopathological parameters with the autoantibody measurements *χ* ^2^‐tests, Fisher's exact tests, and t‐tests were used as appropriate. At baseline, uni‐, and multivariable logistic regression models were performed to assess whether autoantibodies could predict treatment responses, whereby odds ratio (OR) and 95% confidence intervals are reported. To avoid perfect prediction of the outcome in the multivariable analysis at the second blood draw, absolute risk differences (RD) were estimated within a generalized linear model. All multivariable analyses were adjusted for tumor entity only regardless of significance in the univariable analyses in order to account for potential differences in response rates and PFS depending on the tumor type. Moreover, no further variables were included to not violate the 1 in 10 rule.

Kaplan–Meier curves were used to estimate PFS, and the log‐rank test was used to compare groups. Uni‐ and multivariable Cox regression hazard models were implemented. A two‐sided *p*‐value of <0.05 was considered significant for all statistical analyses.

All statistical analyses were performed using Stata for Windows Version 16 (StataCorp LP).

### 2.2 Ethics

Written informed consent was obtained from each patient included in the study. This study was approved by the local ethics committee at the Medical University of Graz (29–593 ex 16/17).

## 3 RESULTS

Overall, 44 patients treated with ICIs were included in this prospective single‐center cohort study and baseline autoantibody levels were measured in all of these. Median follow‐up time was 13.5 (IQR 2.8–25.1) months. The most prevalent cancer entity was NSCLC (*n* = 15), followed by renal cell carcinoma (*n* = 11), head and neck squamous cell carcinoma (*n* = 6), urothelial carcinoma of the urinary bladder (*n* = 7) and colorectal cancer (*n* = 3). One patient had gastric cancer and one cholangiocarcinoma. Most patients were either treated with the PD‐1 inhibitors Nivolumab (*n* = 22) or Pembrolizumab (*n* = 20). In addition, one patient was treated with the PD‐L1‐inhibitor Atezolizumab, whereas one patient received an ICI combination therapy consisting of Nivolumab plus Ipilimumab. Twenty‐seven (61.4%) patients received ICI within a 2nd or 3rd line treatment setting, while 17 (38.6%) patients underwent ICI therapy as 1st line therapeutic approach. Considering all measured autoantibody titers, 21 (47.7%) patients had any positive result of autoantibody measurement. At baseline, 18/44 (40.9%) patients had positive ANA titers, 5 (11.4%) patients had positive ENA measurements, 2 (4.5%) patients had positive hepatopathy antibody titers, and one patient had positive titers of antibodies associated with myositis. Some patients had an overlap between different types and autoantibodies: four patients had both, positive ANA and ENA titers and one patient had both, positive ANA and hepatopathy antibodies. No patients had positive antibody titers associated with rheumatoid arthritis (also see Table S1 for individual autoantibody measurements).

Autoantibodies were not associated with clinicopathological parameters at baseline (Table 1). In detail, the distribution of gender, age, BMI, tumor entity, smoking status, and histology did not significantly differ according to positive or negative measurements of ANA, ENA, hepatopathy‐, and myositis antibodies (all *p* > 0.05).

**TABLE 1:** Summary table of the study population

	*n* (%miss.)	Summary measure	*p*‐value^a^
Demographic variables			
Sex	44 (0%)		0.599
Female		13 (30%)	
Male		31 (70%)	
Age (years)	44 (0%)	63.5 [57–70.5]	0.0571
BMI (kg/m^2^)	44 (0%)	24.4 [21.4–26.6]	0.5435
Cancer entities	44 (0%)		0.612
Non‐small cell lung cancer		15 (34%)	
Adenocarcinoma		9	
Squamous cell carcinoma		5	
Large cell lung carcinoma		1	
Renal cell carcinoma		11 (25%)	
Clear cell		9	
Papillary		1	
Translocation RCC		1	
Head and neck (squamous cell)		6 (14%)	
Bladder cancer		7 (16%)	
Colorectal cancer		3 (7%)	
Gastric cancer (signet ring cell)		1 (2%)	
Cholangiocellular carcinoma		1 (2%)	
History of smoking	44 (0%)	23 (52%)	0.989
PD‐L1 expression	16 (64%)		
Positive		10 (23%)	
Negative		6 (14%)	
Treatment	44 (0%)		0.567
Nivolumab		22 (50%)	
Nivolumab/ipilimumab		1 (2%)	
Pembrolizumab		20 (46%)	
Atezolizumab		1 (2%)	
Treatment line	44 (0%)		0.442
1st line		17 (38.6%)	
2nd line		21 (47.7%)	
3rd line		6 (13.6%)	
Autoantibodies at baseline	44 (0%)		NA
Positive		21 (48%)	
Negative		23 (52%)	

^a^ Association of clinico‐pathological parameters with positive autoantibody screening at baseline.

### 3.1 Association of clinical endpoints with autoantibody levels at baseline

For the entire study population, DCR was 45.5%, whereas ORR was 22.7%. Overall, the best responses were PR in 10 patients, SD in 10 patients and progressive disease (PD) in 24 patients, respectively. There was no individual who experienced CR during the follow‐up period.

**FIGURE 1|:**
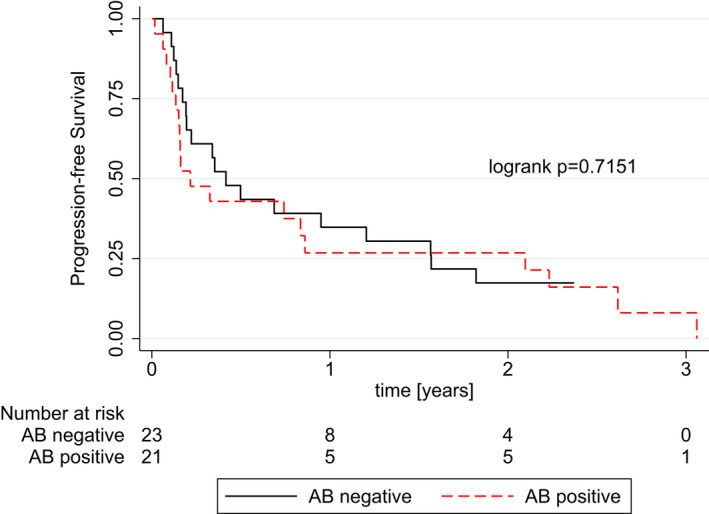
Kaplan–Meier curves showing progression‐free survival (PFS) for patients with positive versus negative autoantibody screening at treatment initiation

Disease control rate differed numerically in patients with and without any positive autoantibody measurement at baseline (52.2% vs. 38.1%), however, this difference was not statistically significant (*p* = 0.349). The objective response rate was similar in patients with and without positive autoantibody titers (19.1% vs. 26.1%, *p* = 0.578). The presence of any autoantibodies at treatment initiation was no statistically significant predictor of DCR, neither in uni‐, nor multivariable analyses adjusted for tumor entity (Table 2). Likewise, patients with positive autoantibody titers did not show different odds of experiencing PR or CR as compared to patients without autoantibodies (Table 2). Moreover, there was no statistically significant difference in PFS between patients with and without any autoantibodies at baseline (log‐rank *p* = 0.7151; Figure 1), and positive autoantibody measurement was not a statistically significant predictor of PFS in the univariable (HR = 1.131, 95% CI 0.584–2.189, *p* = 0.715) or multivariable Cox regression analysis adjusting for tumor entity(HR = 0.944, 95% CI 0.457–1.950, *p* = 0.876) [Table 3].

**TABLE 2:** Univariable and multivariable analyses of autoantibody levels at treatment initiation predicting DCR and ORR. Multivariable analysis was adjusted for tumor type

Variable	Univariable analysis		Multivariable analysis	
	OR (95% CI)	*p*‐value	OR (95% CI)	*p*‐value
DCR				
Any autoantibody				
Negative	1 (reference)		1 (reference)	
Positive	0.56 (0.17–1.88)	0.351	0.62 (0.16–2.37)	0.480
ANA				
<1:80 (negative)	1 (reference)		1 (reference)	
≥1:80 (positive)	0.63 (0.19–2.16)	0.468	0.54 (0.17–2.48)	0.529
ORR				
Any autoantibody				
Negative	1 (reference)		1 (reference)	
Positive	0.67 (0.16–2.79)	0.579	0.79 (0.17–3.63)	0.765
ANA				
<1:80 (negative)	1 (reference)		1 (reference)	
≥1:80 (positive)	0.95 (0.23–4.01)	0.947	1.11 (0.25–4.94)	0.890

Abbreviations: ANA, antinuclear antibody; CI, confidence interval; DCR, disease control rate; OR, odds ratio; ORR, objective response rate.

**TABLE 3:** Uni‐ and multivariate Cox regression regarding PFS at baseline and after 8–12 weeks of therapy. Multivariable analysis was adjusted for tumor type. HR – hazard ratio

Variable	Univariable analysis		Multivariable analysis	
	HR (95% CI)	*p*‐value	HR (95% CI)	*p*‐value
1st blood draw (baseline)				
Any autoantibody				
Negative	1 (reference)		1 (reference)	
Positive	1.131 (0.584–2.189)	0.715	0.944 (0.457–1.950)	0.876
ANA				
<1:80 (negative)	1 (reference)		1 (reference)	
≥1:80 (positive)	0.971 (0.492–1.917)	0.933	0.801 (0.377–1.703)	0.565
2nd blood draw				
Any autoantibody				
Negative	1 (reference)		1 (reference)	
Positive	1.317 (0.567–3.061)	0.522	0.923 (0.343–2.482)	0.874
ANA				
<1:80 (negative)	1 (reference)		1 (reference)	
≥1:80 (positive)	0.834 (0.362–1.922)	0.670	0.405 (0.145–1.138)	0.086
ANA level change				
No increase	1 (reference)		1 (reference)	
Increase	0.825 (0.338–2.011)	0.672	0.614 (0.245–1.540)	0.298

Abbreviations: ANA, antinuclear antibody; CI, confidence interval; DCR, disease control rate; ORR, objective response rate.

Additionally, we analyzed whether the presence of different autoantibody subgroups at baseline might show an association with primary and secondary study endpoints. Since only few patients had positive ENA, hepatopathy‐ or myositis autoantibodies, this subgroup analysis was restricted to ANA titers. Patients with ANA titers ≥1:80 were considered ANA positive. Positive ANA titers at the initiation of ICI treatment were not statistically significantly associated with DCR and ORR in both uni‐ and multivariable analyses adjusted for cancer type (Table 2).

Moreover, there was no difference in PFS between patients with pathological ANA titers as compared to patients with negative ANA titers (log‐rank *p* = 0.933). Likewise, uni‐ and multivariable Cox proportional hazard models did not show any statistically significant association with PFS (Table 3).

Additionally, subgroup analysis for the three most prevalent tumor types in our cohort was conducted. There was no statistically significant difference in DCR in patients with or without any positive autoantibody measurement at baseline in NSCLC (*p* = 0.205), renal cell carcinoma (*p* = 0.898), and urothelial carcinoma of the urinary bladder (*p* = 0.809). Likewise, ORR did not differ according to the presence of any autoantibody at baseline in NSCLC (*p* = 0.792), renal cell carcinoma (*p* = 0.898), or urothelial carcinoma of the urinary bladder (*p* = 0.350).

Considering positive ANA titers only, both DCR and ORR did not significantly differ in NSCLC (*p* = 0.189 and 0.438), renal cell carcinoma (*p* = 0.898 and 0.898) and bladder cancer (*p* = 0.809 and 0.350).

### 3.2 Autoantibodies after 8–12 weeks of ICI therapy

Thirty‐one (70.5%) patients had a second follow‐up blood draw 8–12 weeks after initiation of treatment (time point of first tumor response assessment), while 13 (29.5%) patients either dropped out of the study due to PD, unfitness for further treatment or they had received ICI therapy within less than 8 weeks for any other reason.

At time of first response assessment, 18 (58%) patients had any positive autoantibody titer, of which 14 (45.1%), 4 (12.9%), 3 (9.7%), and 3 (9.7%) patients were ANA, ENA, hepatopathy‐ or myositis autoantibody positive, respectively.

Of patients who initially had negative autoantibody titers at treatment initiation (*n* = 16), 4 patients developed increased titers of any autoantibody after 8–12 weeks of ICI treatment, 3 of which developed ANA titers ≥1:80 whereas one patient eventually had increased hepatopathy autoantibodies. As opposed to this, one patient who initially had ANA titers ≥1:80 at baseline, converted to normal serum levels of ANA at the time of first response assessment. As for the change of ANA titers after 8–12 weeks of ICI treatment, 10 patients (32.4%) showed an increase, and 2 patients (6.5%) had a decrease in ANA levels. Eighteen patients (58.1%) had no change in ANA titers.

Disease control rate was not statistically significantly different between patients with and without positive antibody titers 8–12 weeks after initiation of treatment (55.6% vs. 76.9%, *p* = 0.220). Positive Autoantibodies at the 2nd blood draw were not significantly related to response, as defined by DCR or ORR (Table 4), although borderline significant when adjusted for cancer type (DCR: RD = −0.332 95% CI −0.682 to 0.017, *p* = 0.062). Furthermore, positive ANA titers (≥1:80), as well as an increase in ANA titers between 1st and 2nd blood draw did not show any significant RD for DCR and ORR (Table 4). In addition, the presence of any autoantibody, positive ANA, or an increase of the ANA titer was no statistically significant predictors of PFS (Table 3).

**TABLE 4:** Univariable and multivariable analyses of autoantibody levels at 2nd blood draw predicting DCR and ORR. Multivariable analysis was adjusted for tumor type

Variable	Univariable analysis		Multivariable analysis	
	RD (95% CI)	*p*‐value	RD (95% CI)	*p*‐value
DCR				
Any autoantibody				
Negative	1 (reference)		1 (reference)	
Positive	−0.214 (−0.538–0.111)	0.197	−0.332 (−0.682–0.017)	0.062
ANA				
<1:80 (negative)	1 (reference)		1 (reference)	
≥1:80 (positive)	−0.004 (−0.343–0.334)	0.981	0.110 (−0.268–0.489)	0.567
ANA titer change				
No increase	1 (reference)		1 (reference)	
Increase	−0.05 (−0.419–0.319)	0.790	−0.147 (−527–0.233)	0.449
ORR				
Any autoantibody				
Negative	1 (reference)		1 (reference)	
Positive	−0.693 (−2.810–1.424)	0.521	−0.107 (−0.443–0.229)	0.533
ANA				
<1:80 (negative)	1 (reference)		1 (reference)	
≥1:80 (positive)	−0.067 (−0.395–0.261)	0.688	0.078 (−0.241–0.398)	0.631
ANA titer change				
No increase	1 (reference)		1 (reference)	
Increase	−0.033 (−0.382–0.315)	0.851	0.042 (−0.307–0.391)	0.813

Abbreviations: ANA, antinuclear antibody; CI, confidence interval; DCR, disease control rate; ORR, objective response rate; RD, risk difference.

Since fewer patients had a second blood draw, meaningful subgroup analyses for cancer types were limited to NSCLC and renal cell carcinoma. Patients with NSCLC and no autoantibodies after 8–12 weeks of ICI treatment showed significantly higher rates of response as indicated by DCR (*p* = 0.038) but not as indicated by ORR (*p* = 0.490). In patients with renal cell carcinoma, DCR (all patients responders) and ORR (*p* = 0.850) did not significantly differ depending on the presence of any positive autoantibody at the second blood draw. Results were similar when analyzing the presence of positive ANA titers as well as increase vs decrease in ANA titers after 8–12 weeks of ICI therapy. There was no significant difference in DCR and ORR in patients with NSCLC or renal cell carcinoma (all *p* > 0.05).

### 3.3 Autoantibodies as predictors for immune‐related adverse events

In total, 5 (11.4%) patients of the study cohort developed grade 3 or higher irAEs. Accordingly, liver and kidney laboratory parameters indicative of associated toxicities are reported in supplementary Table 2. Two patients developed severe autoimmune hepatitis, two patients had severe hypophysitis and one patient each developed rash, thyroiditis, or pneumonitis. Patients with higher‐grade adverse events had a significantly higher rate of response as indicated by DCR (*p* = 0.009), whereas there was no significant difference in ORR (*p* = 0.877). Of patients with severe irAEs, two patients had positive autoantibody titers and both were ANA positive at treatment initiation. No patients who developed irAEs, had ENA, hepatopathy‐, myositis‐, or RA autoantibodies at baseline. Patients with positive autoantibody titers demonstrated no increased risk of developing any grade 3 or 4 irAEs (OR = 0.702, 95% CI 0.105–4.674, *p* = 0.714). There was no statistically significant difference regarding the distribution of irAEs in patients with positive ANA titers at baseline (*p* = 0.965) and at the second blood draw (*p* = 0.8). Furthermore, there was no significant difference in patients who had an increase in ANA titers between the first and second blood draws (*p* = 0.410).

## 4 DISCUSSION

Several studies have investigated the role of different autoantibodies and their potential to be used as biomarkers for the assessment of treatment efficacy, survival outcomes, or the risk to develop severe irAEs in patients treated with ICI therapy.^18^, ^19^, ^21^, ^23^, ^24^, ^25^ To the best of our knowledge, the present study is the first prospectively assessed pan‐cancer study considering a wide range of different autoantibodies, including ANA, ENA, hepatopathy‐, rheumatoid arthritis‐, and myositis autoantibodies at two defined longitudinal time points within the course of ICI treatment. Our study did not show any statistically significant relationship between any positive autoantibody measurement at baseline with the clinical endpoints DCR, ORR, and PFS. After 8–12 weeks of ICI treatment, the presence of any pathological autoantibody titer did not predict treatment response as indicated by DCR, ORR, or PFS. Furthermore, ANA levels at both timepoints and the increase in ANA titers were no significant predictors of treatment outcomes. Subgroup analysis stratified by tumor entity did not reveal significant differences in response rates (DCR and ORR) except for DCR in patients NSCLC after 8–12 weeks of ICI treatment. Finally, we did not observe evidence of an association between elevated autoantibody‐ or ANA titers with the occurrence of grade 3 or higher irAEs.

To date, the majority of published studies that evaluated different autoantibodies as biomarkers for safety and efficacy of ICI treatment were conducted in advanced or metastatic NSCLC patients.^18^, ^21^, ^22^, ^23^, ^24^, ^25^ Two studies considered additional autoantibodies besides ANA. Toi et al.^23^ retrospectively analyzed 137 patients with NSCLC and found out that individuals with preexisting antibodies (ANA, rheumatoid factor, antithyroglobulin, antithyroid peroxidase) at treatment initiation had more favorable outcome as indicated by PFS as compared to patients without antibodies.^23^ Moreover, patients with preexisting antibodies showed higher response rates, yet ANA, rheumatoid factor, or antithyroid antibodies were not individually associated with ORR and DCR.^23^ Likewise, Giannicola and colleagues^18^ reported that positive autoantibodies (ANA, ENA, anti‐smooth muscle cell antigens), emerging within the first 30 days of ICI treatment, were associated with increased PFS and overall survival (OS) in NSCLC patients receiving Nivolumab therapy. Both mentioned studies did have a retrospective study design, which should be noted as an important limitation in terms of a potential selection bias. In contrast, in our prospective study, the presence of any autoantibody or ANA positivity was, if anything, numerically associated with lower DCR and ORR in both uni‐ and multivariable analyses, although this did not receive statistical significance with the number of patients and events we had.

Antinuclear antibodies represent a class of autoantibodies against cellular components in the nucleus of a cell and have been repeatedly reported in malignant diseases besides autoimmune diseases, such as, but not limited to systemic lupus erythematous, Sjögren's syndrome, and other connective tissue diseases.^22^ The presence of autoantibodies indicates auto‐reactive B‐cells. When focusing on the role of ANA in ICI treatment alone, previously reported results are conflicting. Sakakida et al.^19^ evaluated ANA titers in 191 patients with different cancer entities who received ICI treatment. In this analysis, no statistically significant relationship with DCR, ORR, and PFS could be observed, which is in line with the results of our present study. These results corroborate the previously discussed results by Toi et al.,^23^ who could not detect differences in DCR, ORR, and PFS in patients with preexisting ANA. Likewise, Mouri et al.^25^ found no significant relationship between ANA positivity and survival in 266 NSCLC patients, although PFS was numerically higher in patients with positive ANA. In contrast, studies by Morimoto et al^21^ and Yoneshima et al.^24^ including 77 and 83 patients with advanced NSCLC, respectively, reported significantly shorter PFS and OS in patients with positive ANA titers. Nonetheless, the differences in analyzed autoantibodies and varying cut‐offs for positive ANA titers are difficult if not impossible to compare. Notably, Sakakida et al.,^19^ as well as Morimoto et al.^21^ defined ANA positivity as ANA titers ≥1:160, resulting in considerably less patients being classified as ANA positive as compared to other published studies on this research topic.^23^, ^24^, ^25^ With the definition of ANA positivity at ANA titers ≥1:80 our study is consistent with previously used cut‐offs that are ranging between 1:40 and 1:160. Considering the diverging results of previous studies, as well as their retrospective study design, our prospective longitudinal study adds important information regarding the potential role of ANA in the course of ICI treatment.

Finding highly reliable risk factors and biomarkers for the development of irAEs represents an extremely important and clinically relevant question in order to adequately monitor cancer patients prone to develop severe and potentially even life‐threatening irAEs.^16^ Various autoantibodies have been suggested as potential biomarkers of irAEs, such as myositis (anti‐acetylcholine receptor antibodies),^27^ thyroiditis (antithyroglobulin, antithyroid peroxidase),^23^, ^28^ hypophyitis (anti‐GNAL, anti‐ITM28),^29^ pneumonitis (anti‐CD74),^29^ or skin reactions (anti‐BP180).^30^ However, strong and robust evidence of whether the presence of autoantibodies might be associated with the development of severe irAEs is still not clear. Sakakida et al.^19^ observed a higher frequency of positive ANA in patients who developed colitis, although ANA was not associated with irAEs of any grade, corroborating results by Mouri et al.^25^ Conversely, Morimoto et al.^21^ reported a higher discontinuation rate of treatment due to severe adverse events in the ANA positive group. Interestingly, in the study by Toi et al.^23^ the frequency of any irAEs was significantly higher in patients with any preexisting antibodies or preexisting rheumatoid factor, yet, there was no statistically significant difference in the frequency of grade 3 or higher irAEs.^23^ In the present prospective study, we did not observe an increased frequency of grade 3 or higher irAEs in patients with any positive autoantibody, positive ANA titers, or an increase in ANA titers after ICI treatment.

Finally, our study aimed to assess the longitudinal evolution of autoantibody levels during ICI treatment and the potential relationship of these dynamic changes with treatment response and irAEs. This showed that 4 patients changed from negative to positive autoantibody measurement and 10 patients showed an increase in ANA titers over time consistent with autoantibody induction by ICIs. In contrast, one patient changed from positive to negative and 2 patients had a decrease in ANA levels consistent with longitudinal variability in the measurements. These data indicate that ICI treatment may lead to autoantibody induction over time.

Some important limitations of our study have to be considered. First, despite the prospective study design, selection bias cannot be entirely excluded due to the inclusion of patients from a single tertiary referral center. Second, due to the relatively small sample size of our patient cohort subgroup analysis stratified for cancer entities should be interpreted with caution. Despite our study has some limitations in sample size, we observed no signals in these 44 patients. However, if any effect on autoantibodies with immunotherapy is detectable, the effect size is very small and then of questionable clinical relevance. Thus, we think our results are meaningful for further prospective studies. Third, due to a pan‐cancer study design, follow‐up protocols and ICI treatment dosing schemes may vary depending on cancer entity. Fourth, as PD‐L1 expression was for the most parts only routinely assessed in NSCLC, unfortunately, PD‐L1 expression status is missing in most patients. The PD‐L1 status plays almost no role in our cohort, as for most of the included patients: renal cell carcinoma, 2nd line urothelial cancer, 2nd line lung cancer treated with nivolumab, and 2nd line head and neck cancer treated with nivolumab—no recommendation of testing the PD‐L1 status has been made by approval status or guidelines. Fifth, lower‐grade irAEs were not monitored in our study, thus no conclusion on the impact of autoantibodies on lower‐ and any‐grade irAEs can be drawn.

Considering the results of our study, the presence of autoantibodies (including ANA, ENA, hepatopathy‐, and myositis autoantibodies) in the serum of cancer patients at baseline, as well as 8–12 weeks after ICI treatment initiation, is not associated with an increased or decreased treatment efficacy, as indicated by DRC, ORR, and PFS. In addition, patients with preexisting or positive autoantibody titers after treatment initiation do not seem to have a higher risk of experiencing higher grade irAEs.

## CONFLICT OF INTEREST

None of the contributing authors have any conflicts of interest, including specific financial interests and relationships and affiliations relevant to the subject matter or materials discussed in the manuscript.

## AUTHOR CONTRIBUTIONS

MP, MS, and DB contributed to the conception and design of the study. SS organized the database. DB performed the statistical analysis. All authors contributed to the interpretation of the results. DB wrote the first draft of the manuscript. All authors contributed to manuscript revision, read, and approved the submitted version.

## ETHICS STATEMENT

Written informed consent was obtained from each patient included in the study. This study was approved by the local ethics committee at the Medical University of Graz (29–593 ex 16/17).

## Supporting information


Appendix S1.

## Data Availability

The dataset for this study is not publicly available by request of the local ethics committee in order to protect the anonymity of the patients.
