# Supplementary material for: Evaluation of autoantibodies as predictors of treatment response and immune‐related adverse events during the treatment with immune checkpoint inhibitors: A prospective longitudinal pan‐cancer study
Source: Cancer Med. 2022 Mar 16;11(16):3074–83. doi: 10.1002/cam4.4675 (PMC9385593; doi:10.1002/cam4.4675)
Supplement: Supplementary file 1 — Appendix S1. [file CAM4-11-3074-s001.docx]

**Supplementary Table 1: Autoantibody measurements at baseline and after 8-12 weeks of ICI treatment**

|  | Autoantibody | 1^st^ blood draw (n=44)  Median [IQR] | Number of Patients positive at 1^st^ blood draw | 2^nd^ blood draw  (n=31)  Median [IQR] | Number of Patients positive at 2^nd^ blood draw |
| --- | --- | --- | --- | --- | --- |
| Antinuclear antibodies | ANA | 0 [0-160] Maximum 5120 | 18 | 0 [0-640]  Maximum 5120 | 14 |
| extractable nuclear antigens  antibodies | ENAscreen | 0.1 [0.1-0.2] | 2 | 0.1 [0.1-0.2] | 2 |
|  | CENPB | 0.2 [0.1-0.3] | 1 | 0.3 [0.2-0.4] | 1 |
|  | dsDNA | 0.9 [0.5-1.5] | 0 | 1 [0.4-1.6] | 0 |
|  | anti-La | 0.1 [0-0.3] | 0 | 0.1 [0-0.3] | 0 |
|  | anti-PM100 | 1 [0-2] | 1 | 1 [0-2] | 1 |
|  | anti-PM75 | 1 [1-2] | 1 | 2 [1-2] | 1 |
|  | anti-RNP70 | 0.1 [0-0.2] | 0 | 0.1 [0-0.3] | 0 |
|  | anti-Ro | 0.2 [0.1-0.3] | 1 | 0.3 [0.2-0.4] | 1 |
|  | anti-SCL70 | 0.0 [0-0.8] | 0 | 0.6 [0-0.8] | 0 |
|  | anti-U1RNP | 1.2 [0.7-1.6] | 0 | 1.3 [1.0-1.8] | 0 |
| Rheumatoid arthritis associated | anti-CPA | 0.8 [0.6-1.25] | 0 | 1.0 [0.7-1.5] | 0 |
|  | Rheumatoid factor IgA | 0 [0-0] | 0 | 0 [0-0] | 0 |
| Hepatopathy-associated | anti-GP210 | 0 [0-0] | 0 | 0 [0-0] | 0 |
|  | anti-LKM1 | 0 [0-0] | 0 | 0 [0-0] | 0 |
|  | anti-M2 | 0 [0-0] | 1 | 0 [0-0] | 1 |
|  | anti-SP100 | 0 [0-0] | 1 | 0 [0-0] | 1 |
|  | anti-SLA-LP | 0 [0-0] | 0 | 0 [0-0] | 1 |
|  | anti-LC1 | 0 [0-0] | 0 | 0 [0-0] | 0 |
|  | anti-F-Actin | 0 [0-0] | 0 | 0 [0-0] | 0 |
| Myositis-associated | anti-EJ | 1 [1-1] | 0 | 1 [1-2] | 0 |
|  | anti-JO1 | 0.1 [0-0.2] | 0 | 0.1 [0-0.2] | 0 |
|  | anti-Ku | 0.5 [0-1] | 0 | 1 [0-2] | 0 |
|  | anti-MDA5 | 2 [1-2] | 0 | 2 [1-2] | 0 |
|  | anti-MI2a | 2 [1-2] | 0 | 2 [2-3] | 0 |
|  | anti-MI2b | 2 [2-3] | 0 | 2 [2-4] | 1 |
|  | anti NXP2 | 2 [1-2] | 0 | 2 [1-2] | 0 |
|  | anti-Oj | 1 [1-2] | 0 | 1 [1-2] | 0 |
|  | anti-PL-12 | 1 [1-2] | 1 | 1 [1-2] | 1 |
|  | anti-PL-7 | 1 [1-2] | 0 | 1 [1-2] | 1 |
|  | anti-SAE | 1 [1-2] | 0 | 1 [1-3] | 0 |
|  | anti-SRP | 2 [1-3] | 1 | 2 [2-4] | 0 |
|  | anti-TIF-1γ | 1 [1-2] | 0 | 1 [1-2] | 0 |

**Supplementary Table 2: Kidney and liver toxicity parameters at baseline and after 8-12 weeks of immunotherapy**. IQR – interquartile range; GGT – gamma glutamyl transferase; AP – alkaline phosphatase; AST – aspartate aminotransferase, ALT – alanine aminotransferase

| Laboratory value | 1^st^ blood draw (n=44)  (median [IQR]) | 2^nd^ blood draw (n=31)  (median [IQR]) |
| --- | --- | --- |
| Creatinine (mg/dL) | 0.90 [0.75-1.17] | 0.95 [0.69-1.2] |
| GGT (U/L) | 59 [27-127] | 46 [23-92] |
| AP (U/L) | 95 [67-130] | 91 [63-139] |
| AST (U/L) | 26 [20-31] | 25 [20-40] |
| ALT (U/L) | 21 [12-36] | 16 [11-28] |
